# Progress in the study of FOXO3a interacting with microRNA to regulate tumourigenesis development

**DOI:** 10.3389/fonc.2023.1293968

**Published:** 2023-10-27

**Authors:** Liying Sun, Jiaqi Liu, Dongbo Bao, Cheng Hu, Yundong Zhao, Shuang Chen

**Affiliations:** ^1^ College of Laboratory Medicine, Jilin Medical University, Jilin, China; ^2^ College of Medical Technology, Beihua University, Jilin, China

**Keywords:** microRNA, FOXO3a, interaction, signalling pathway, tumour therapy/diagnosis

## Abstract

FOXO3a is a protein of the forkhead box family that inhibits tumour cell growth. One of the regulatory modes affecting the role of FOXO3a is microRNA targeting and degradation of its mRNA expression, and conversely, aberrant expression of FOXO3a as a transcription factor also influences microRNA levels. We summarized the results of the regulatory interactions of twenty-five microRNAs with FOXO3a in five types of malignant tumours and found that dual microRNAs synergize with FOXO3a to inhibit breast cancer cell growth including two groups; Three individual microRNAs collaborated with FOXO3a to restrain hepatocellular carcinoma progression; Twelve individual microRNAs antagonized FOXO3a to promote the development of a single tumour cell, respectively; and five microRNAs antagonized FOXO3a to contribute to the progression of more than two types of tumours. The above findings demonstrated the tumour suppressor effect of FOXO3a, but another result revealed that miR-485-5p and miR-498 inhibited the growth of hepatocellular carcinoma cells by antagonizing FOXO3a when acting in combination with other long-stranded non-coding RNAs, respectively, suggesting that FOXO3a at this moment plays the function of promoting the tumour progression. The PI3K/AKT, Snail, VEGF-NRP1, and Wnt/β-catenin signalling pathways perform crucial roles in the above process. It is anticipated that the above studies will assist in understanding the effects of FOXO3a-MicroRNA interactions in cancer genesis and development, and provide new perspectives in the treatment of malignant tumours.

## 1 Introduction

Tumour development involves a variety of factors, such as suppression of anti-tumour immune mechanisms and activated expression of tumour-related genes. Early identification of oncogenes and new targets is important for cancer diagnosis and treatment and also contributes to the development of new drugs (1, 2). FOXO3a, as a tumour-suppressor gene, is phosphorylated by kinases and then transferred to the cytoplasm via a signalling pathway, and degraded by ubiquitination (3). In recent years, research on malignant tumours has focused on the genetic level. It has also been found that non-coding RNAs have antagonistic or synergistic effects with FOXO3a in tumour cells. Non-coding RNAs, especially microRNAs, are highly correlated with tumour growth, and thus have increasingly become the object of research. To date, thousands of microRNAs have been identified (4, 5), providing molecular indicators for the diagnosis and treatment of cancer. The combined effects of FOXO3a and microRNAs in tumours have also attracted significant attention, and the roles of these two factors in different tumours are also diverse. Therefore, this review explores the interactions between FOXO3a and microRNAs in multiple malignant tumours, aiming to provide a new perspective for the identification of oncogenes and the development of antitumor drugs.

## 2 The structure of FOXO3a

**Figure 1:**
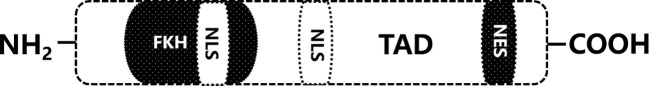
The functional domain of FOXO3a. FKH, DNA binding domain; NLS, nuclear localization signal; TAD, trans-activation domain; NES, nuclear export signal.

Forkhead box proteins constitute a group of evolutionarily conserved proteins with a DNA-binding domain consisting of three α-helices and two winged helical folds. Since different subtypes of FOX proteins show diverse binding specificities and cellular effects, the FOX family can be grouped into four different subfamilies, FOXM, FOXK, FOXA, and FOXO, on the basis of additional structural domains and conserved sequences. The FOXO family is involved in various cellular processes, such as apoptosis, oxidative stress, and cell cycle progression (6), with FOXO1, FOXO3a, FOXO4 and FOXO3a being more extensive; among these proteins, FOXO3a has been described in the most detail in the literature. FOXO3a, encoded by a gene located on human chromosome 6q21, is approximately 72 kDa in size and consists of 673 amino acid residues (7). It carries a highly conserved transcription element, and five structural domains (**Figure 1**): two nuclear localization signals (NLSs) and a nuclear export signal (NES) distant from the NLSs, contributing to the bidirectional movement of FOXO3a between the nucleus and the cytoplasm; a trans-activating structural domain (TAD) close to the carboxyl terminus, which allows direct and indirect activation of FOXO3a target gene expression; and a highly conserved forkhead DNA-binding domain (FKH), which the site of interaction with a candidate gene (8).

## 3 The interplay of FOXO3a with microRNAs

**Figure 2:**
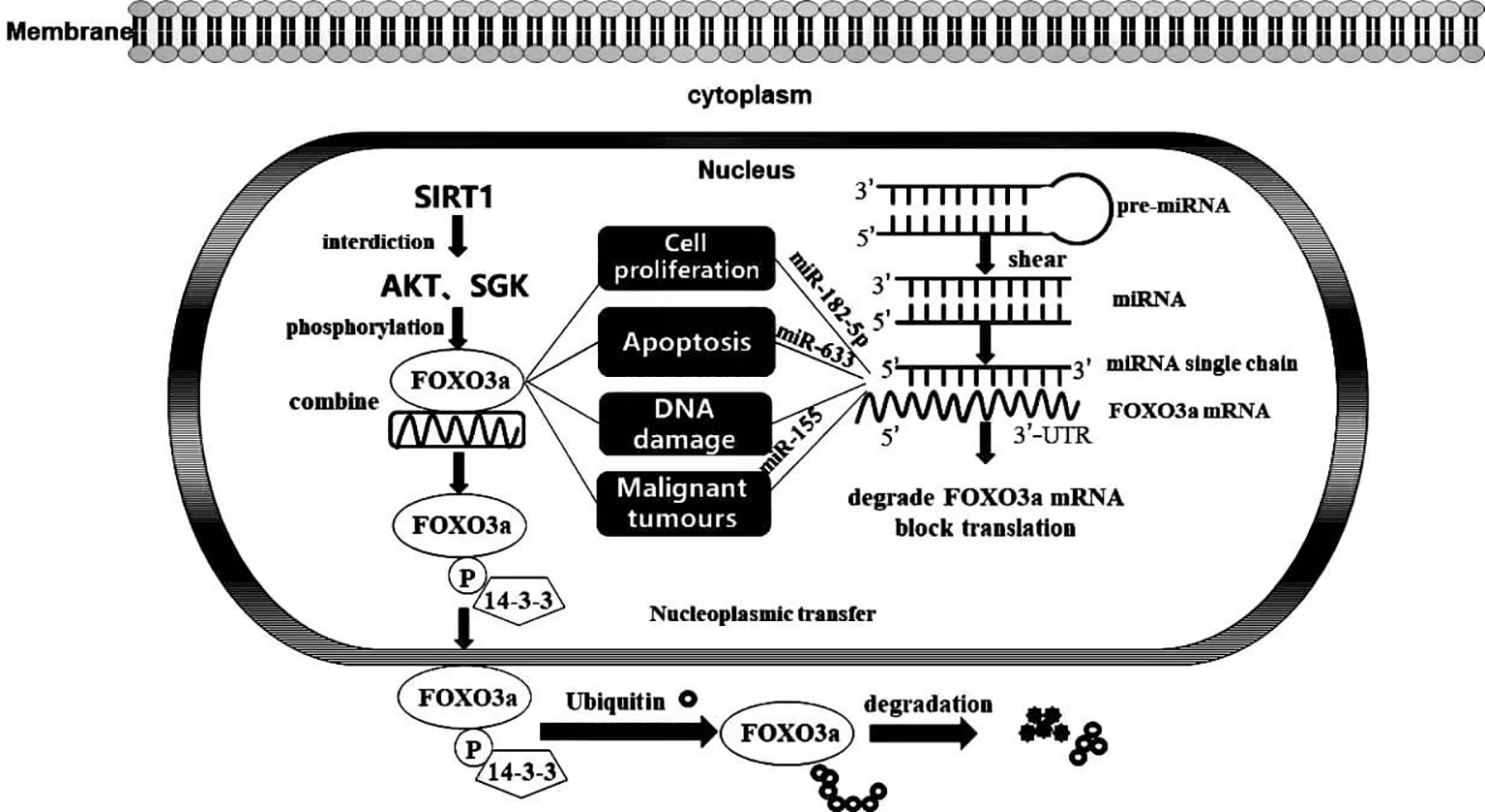
FOXO3a transcription factor activity and its interaction with MicroRNA. Activation and inactivation processes of FOXO3a transcription factors in cells and the impacts of MicroRNA targeting on FOXO3a on biological progression.

In general, FOXO3a exerts an influence in the nucleus to induce the transcription of target genes; simultaneously, it may respond to extracellular stimuli, such as drugs and UV radiation. Phosphokinase AKT and glucocorticoid-inducible kinase SGK prompt FOXO3a phosphorylation, which then binds to 14-3-3 protein, blocking access to an NLS in FOXO3a, driving its localization to the cytoplasm. Ultimately, FOXO3a is ubiquitinated and degraded in the cytoplasm in a proteasome-dependent manner, and deacetylation of the silencing regulatory protein SIRT1 diminishes FOXO3a phosphorylation and degradation, enabling FOXO3a to bind stably to target gene DNA. With the continuous advancement in genomics techniques, microRNAs have been shown to be steadily expressed in tumour tissues and to regulate the expression levels of genes by interacting with the 3’-untranslated region (3’-UTR) of an mRNA, decreasing its susceptibility to degradation and ultimately facilitating the negative regulation of mRNA levels to modify protein content (9). Notably, microRNAs might regulate the origin and progression of multiple conditions, including cancer, by targeting the mRNA of FOXO3a (**Figure 2**). In addition, our group previously showed that FOXO3a expression induces autophagy and apoptosis in breast cancer cells. Moreover, apoptosis is tightly connected with malignant tumour evolution, and the potential effect of a strategy to moderate FOXO3a activity through an anti-microRNA mechanism that promotes apoptosis is being increasingly recognized. Posttranscriptional therapy mediated by specifically targeting microRNAs facilitates the critical role played by individualized medicine. Nevertheless, the interaction between a microRNA and FOXO3a is not limited to apoptosis induction; therefore, elucidating the mechanism underlying tumorigenesis at the molecular level is of profound importance for the diagnosis and precise treatment of disorders.

### 3.1 Functions of FOXO3a and microRNAs in breast cancer

Breast cancer is among the most frequently diagnosed cancers in females (10, 11). A range of medical treatments, of which surgery, radiotherapy and chemotherapy have been established, can exert a profound impact on women’s physical and psychological well-being by causing cellular damage to healthy tissues; therefore, multiple and interrelated risk factors, especially genetic and epigenetic factors, have been major foci of research in the past few years (12). Molecules, especially those at the genetic level, related to microRNA action are at the centre of tumour diagnosis and show great therapeutic promise. Considering the biological role of targeted microRNAs that block transcription and delay translation, we sought to describe its powerful inhibitory effect on cell proliferation, and FOXO3a, as a focus of tumour research, clearly interacts with microRNAs, making it another key therapeutic candidate to review.

#### 3.1.1 MicroRNA impacts the role of FOXO3a

MiR-182-5p and miR-940 are oncogenes in breast cancer tissues and cells, respectively, and are associated with cancer pathogenesis. Overexpression of miR-182-5p and miR-940 in tumour tissues and cells represses FOXO3a 3’-UTR, which in turn affects its expression level, and previous findings have implicated these microRNAs in breast cancer prognosis and oncogene epigenetic (13, 14). MiR-21 is among the most frequently expressed oncogenic microRNAs, and as I have also observed, its role in breast cancer was first reported by Lorio (15). In more than one article, miR-34 family members have also been identified as inhibitors in several types of cancer (16), and Liu et al. demonstrated for the first time that in p53-mutation-inactivated breast cancer cell lines, miR-21 regulated miR-34b/c expression through the PTEN/AKT/FOXO3a signalling pathway. When miR-21 levels are reduced, leading to enhanced PTEN expression, PTEN inhibits AKT activity, and the loss of AKT action results in FOXO3a retention in the nucleus without being phosphorylated. This nucleus-retained FOXO3a drives miR-34b/c upregulation, and miR-34b/c exerts a stronger antitumour effect when coinjected with inhibitors of miR-21 than when used alone as a treatment (17). MiR-34b/c, which is regulated by FOXO3a, exerts anti-tumour effects by actively counteracting the oncogenic effects of miR-21. This shows that exploring microRNAs that can be used as therapeutics together with drugs will produce better results. Breast cancer cells consume a large amount of energy derived from glucose metabolism in cells in a hypoxic state, and it has been proven that FOXO3a inhibits the glycolytic process (18). Kim et al. demonstrated that miR-155 increased the glucose metabolism rate and mediated breast cancer cell expansion by targeting FOXO3a to regulate the cMYC oncogene *in vivo* and in deletion mouse models of breast cancer; similar results have been obtained with human triple-negative breast tumour cells (19). VEGF is a dimeric glycoprotein secreted by a number of cell types, including endothelial cells and smooth muscle cells, and is the primary effector of the angiogenic pathway. Investigations into FOXO3a targeting VEGF to inhibit tumour angiogenesis have been published (20). Experimental studies have proven that FOXO3a interacts with the miR-29b/miR-338 promoter to inhibit breast cancer cell proliferation, while miR-29b and miR-338 target the 3’-UTR of VEGF-A and NRP1, respectively, to oppress breast cancer cell growth. Thus, the FOXO3a-miR-29b/miR-338-VEGF-A/NRP1 axis is suggested to be abnormally regulated and to play a crucial role in the development of breast cancer (21). Moreover, overexpressing all three factors in breast cancer is thought to boost the therapeutic benefits of each factor.

#### 3.1.2 FOXO3a induces changes in microRNA levels

Similar to the previous example where miR-34b/c was shown to play roles, some microRNA actions are modified by FOXO3a. The epithelial-mesenchymal transition plays a pivotal role in breast cancer cell invasion and metastasis, and clarifying the regulatory mechanism of this transformation response will greatly help in gaining greater insight into the related mechanisms, enabling measures for blocking further breast cancer progression to be identified. Overexpressed FOXO3a in breast cancer cell lines leads to a decline in the invasive and migratory capabilities of breast cancer cells and to the considerable inhibition of miR-10b expression. Measurements of the expression of the epithelial-mesenchymal markers E-cadherin and N-cadherin showed that when the FOXO3a level was increased, the metastasis of breast cancer cells was inhibited (22). The aforementioned *in vitro* and *in vivo* studies revealed a complex relationship between microRNAs and FOXO3a, and the interaction between these two factors was found to be essential to the behaviour of malignant tumours. In future research, we will consider the relationship between specific microRNAs and FOXO3a as a strategy to develop new avenues to breast cancer treatment.

### 3.2 The role played by FOXO3a and microRNAs in hepatocellular carcinoma

Liver cancer is one of the most prevalent types of cancer, showing the highest mortality rate among the top five malignant tumours (23). Despite the maturity of liver cancer treatment, more than one-half of patients experience recurrence and distant metastases after surgical resection (24). It is difficult for medical practitioners to identify reliable therapeutic targets and develop targeted drugs to keep liver cancer patients free of disease. An increasing number of studies have demonstrated that microRNA expression levels correlate with the clinicopathological characteristics of liver cancer patients and are expected to become biomarkers of liver cancer prognosis in the future (25, 26). However, research on microRNAs is still in the exploratory stage, and the suggested associations of many microRNAs with liver cancer factors have not been fully validated.

#### 3.2.1 MicroRNA acting on FOXO3a induces tumorigenesis

AKT, a classical signal transduction factor, regulates cell growth through its action in phosphorylating other proteins in its pathway, and a study showed that when overexpressed, miR-182-5p reduced the level of FOXO3a to promote the proliferation of cancer cells via activation of the AKT signalling pathway and the downstream targets Bcl-2 and Bcl-xl (27). miR-182-5p also induced the activation of the Wnt signalling pathway and the downstream targets c-Myc and cyclin D1. Wnt signalling plays a role in maintaining cell mitosis and stem cell renewal, indicating that the upregulation of FOXO3a expression played a significant role in inhibiting the progression of hepatocellular carcinoma by suppressing the kinase expression level, and research has shown that FOXO3a is an important interfering factor in the malignant behaviour of hepatocellular carcinoma. MiR-3682-3p directly targets and inhibits FOXO3a expression, and FOXO3a level reduction enhances the downstream signalling pathway PI3K/AKT/β-catenin/c-Myc. c-Myc in turn induces miR-3682-3p expression, forming a PI3K/AKT/β-catenin/c-Myc/miR-3682-3p positive feedback loop that promotes the stemness of hepatocellular carcinoma cells (28). FOXO3a is a clear tumour suppressor gene that regulates the expression of downstream proapoptotic factors such as p27Kip1 and Bim (29, 30). It exerts antitumour properties, such as the inhibition of cell proliferation and the promotion of apoptosis. Various studies have been conducted to investigate the potential functions of FOXO3a in hepatocarcinogenesis, and miR-155 has been found to inhibit the apoptosis of hepatocellular carcinoma cells by inhibiting FOXO3a action, thereby reducing the activity of the downstream proapoptosis protein Bim and weakening the activity of caspase-9 and caspase-3 (31). ING4 is an integral protein in the ING family and inhibits tumour cell proliferation (32). ING4 suppresses miR-155 targeting FOXO3a mRNA while triggering the FOXO3a-transacting target Bim to increase the apoptosis of hepatocellular carcinoma cells, and miR-155 mimics decreases the FOXO3a content (33). Clearly, when FOXO3a is activated via a decrease in the level of regulatory miR-155, the apoptosis rate of tumour cells is increased, and the survival rate of cancer cells is reduced. Several of these findings reveal one of the factors responsible for the rapid growth of malignant cells in liver cancer *in vivo*, and the massive expression of oncogenic microRNAs predicts a poor prognosis, while the key steps to curb tumour progression and improve prognosis are to induce apoptosis of malignant cells and intervene in the expression of oncogenic genes.

#### 3.2.2 Effect of the microRNA-induced negative regulation of FOXO3a activity on drug resistance in hepatocellular carcinoma

In the past decade, the rational combination of targeted drugs with conventional doxorubicin has become a promising approach to the treatment of hepatocellular carcinoma, and deeper insight into the molecular mechanisms of the drug has been reported (34). Autophagy is a self-protective catabolic process induced by cellular stress and is regarded as a mechanism that can reverse drug resistance in cells (35). Studies have shown that overexpressed miR-223 inhibits doxorubicin-induced autophagy, which leads to the susceptibility of cells to doxorubicin resistance in cells, and FOXO3a plays an essential mediating role in the induction of autophagy in hepatocellular carcinoma. A miR-223 overexpression optimization model for clinical application deserves further study (36). Acquired resistance to sorafenib, a multikinase inhibitor used to treat advanced hepatocellular carcinoma, is a primary obstacle to the effective treatment of hepatocellular carcinoma, and increasing the rate of apoptosis may fundamentally address the problem of drug resistance. Dong et al. proved that miR-124-3p.1 targeted AKT2 and SIRT1 to regulate the phosphorylation and deacetylation of FOXO3a, sensitizing hepatocellular carcinoma cells to sorafenib-induced apoptosis while increasing FOXO3a activity in cancer therapy (37).

#### 3.2.3 Variations caused by the positive regulation of FOXO3a mediated by microRNAs

The aforementioned studies based on microRNA-targeted FOXO3a in hepatocellular carcinoma cells demonstrated a functionally opposite relationship between the microRNAs and the protein; however, a study closely related to metabolic reprogramming showed divergent findings. When the oxygen level is low, the efficiency of glycolysis in cancer cells is strengthened, supplying the energy needed for tumour growth, an outcome known as the Warburg effect (38). The P protein (HBp) encoded by the HBV virus interacts with FOXO3a to promote the activity of miR-30b-5p in HBV-positive hepatocellular carcinoma, and MINPP1 inhibits tumour cell proliferation. In the study reporting these findings, a new tumorigenic axis, HBp/FOXO3a/miR-30b-5p/MINPP1, was discovered (39), and it facilitated the regression of HBV-positive hepatocellular carcinoma through a glycolytic bypass that is specific to HBV-associated hepatocellular carcinoma. MINPP1 decreases the number of hepatocellular carcinoma cells via this glycolytic bypass, and it is believed that, in the future, this molecule will emerge as a synergistic drug target for hepatocellular carcinoma therapeutics. Additional results from this study indicated that the overexpression of miR-498 significantly reduced hepatocellular carcinoma cell survival. miR-498 did not inhibit the expression of FOXO3a in hepatocellular carcinoma, despite carrying a binding site that is complementary to that in the FOXO3s 3’-UTR (40); this finding is substantially different to that reported in most prior studies. Thus, it has been suggested other noncoding RNAs such as long noncoding RNAs or circular RNAs in the cell function as competitors to microRNAs and thus facilitate mRNA escape from microRNA-mediated inhibition. Moreover, FOXO3a has been proposed to combine with and induce the transcription of the long noncoding RNA LOC554202 promoter region, and this finding was confirmed by experiments showing that FOXO3a contributes to hepatocellular carcinoma progression by upregulating LOC554202 expression; both FOXO3a and LOC554202 thus promoted tumour cell growth. In contrast, miR-485-5p, one of the microRNAs adsorbed by LOC554202, targets and regulates FOXO3a, exerting a completely opposite oncogenic effect to that exerted by LOC554202 (41). Another long noncoding RNA, PRR34-AS1, suppressed apoptosis, functioned as a sponge of miR-498 and subsequently enhanced the expression of FOXO3a in hepatocellular carcinoma cells (42). With the investigation of malignancies at the genetic level, increased attention has been directed to long noncoding RNAs as microRNA sponges, preventing the microRNAs from binding to mRNAs. Notably, FOXO3a did not exhibit a hepatocellular carcinoma cell proliferation-inhibiting effect in the last two papers. Further reports have established that FOXO3a expression was significantly elevated in patients with hepatocellular carcinoma (43). Thus, the protective function of the body may have led to an increase in the FOXO3a level, which inhibited further development of hepatocellular carcinoma. In an alternative explanation, the inhibitory action of miR-498 in hepatocellular carcinoma was possibly too vigorous, and thus attenuates the tumour-suppressing effect of FOXO3a, in addition, possibly due to the presence of long noncoding RNAs that interfered with FOXO3a expression through some unknown mechanism. Nevertheless, most of the aforementioned experimental findings prove a strong inhibitory effect of FOXO3a on hepatocellular carcinoma progression. Additionally, based on the above review, it is reasonable to think that other pathways affect FOXO3a in hepatocellular tumour cells, particularly those in which long noncoding RNA is highly expressed; thus, within a certain concentration range, FOXO3a expression follows the same direction of change and exerts the same biological effect as long noncoding RNA; however, the specific reasons for these findings need to be identified and confirmed via additional analysis.

### 3.3 Effects of FOXO3a and microRNA in gastric cancer

Gastric cancer is extremely difficult to recognize at an early stage, leading to the annual diagnosis of more than 1 million patients (44), and resistance to conventional therapeutic drugs has made it impossible to achieve a breakthrough in the treatment of gastric cancer patients, making prevention and treatment urgent tasks. The PI3K/AKT signalling pathway, which regulates a variety of tumours, has been the subject of medical research and can directly modulate downstream targets to mediate cell proliferation, apoptosis, and metastasis. Research on this signalling pathway has led to the exploration of specific targets from multiple perspectives, with the results serving as theoretical bases for the diagnosis and treatment of gastric cancer.

#### 3.3.1 microRNA induced alterations in FOXO3a levels

In a survey of the malignant progression of hepatopulmonary metastasis in gastric cancer, miR-582 was reported to inhibit FOXO3a expression through complementary targets, thereby contributing to the PI3K/AKT/Snail signalling pathway to accelerate hepatopulmonary metastasis, and similar results were obtained with mice (45). In addition, miR-122-5p targeting FOXO3a was identified in gastric cancer cells with elevated methemoglobin levels, which prevented apoptosis and promoted tumour progression but exerted no effect on cell migration and cell invasion ability. Clearly, increased FOXO3a expression plays a proapoptotic role, and once FOXO3a levels are reduced, the apoptosis rates in tumour cells decrease, which apparently represents a continuation of cancer cell progression. These studies established a formidable theoretical basis for future clinical drug development, and the drug research process that depends on FOXO3a action is worthy of further development (46). Dual luciferase reporter assays can verify a targeted relationship between microRNAs and FOXO3a, and the significance of multiple microRNAs such as miR-372, miR-96-5p, miR-629 and other microRNAs targeting and mediating FOXO3a activity in gastric cancer cell proliferation and development or mediating cisplatin resistance has been proven (47–49).Moreover, microRNAs foster tumour progression and play an active role in the acquisition of a cisplatin-resistant phenotype. Designing therapeutic regimens targeting microRNAs and augmenting FOXO3a activity may be a promising method for treating patients with cisplatin-resistant gastric cancer. By examining cisplatin-resistant gastric cancer cells, He et al. revealed that overexpression of miR-25 significantly accelerated cell cycle progression and diminished the sensitivity of gastric cancer cells to cisplatin. p27Kip1 is a negative cell cycle regulator that arrests cell cycle progression, prevents the activation of the cyclin-dependent kinase CDK complex, and participates in a variety of physiological and pathological processes as a transcriptional target of FOXO3a. Transfection of miR-25 mimicked the suppressed expression of FOXO3a while lowering the level of p27Kip1, a transcriptional target of FOXO3a, which in turn promoted the growth of drug-resistant cells in gastric cancer (50).

#### 3.3.2 Variations in microRNA levels induced by FOXO3a

Notably, microRNA levels can be regulated by FOXO3a action, and miR-633 plays a critical role in mediating drug resistance in gastric cancer. FOXO3a, a transcription factor that can be localized to the nucleus, represses miR-633 transcriptional activity, when the FOXO3a is overexpressed, and increases the levels of Fas-associated death domain protein FADD, thus activating the apoptotic pathway (51). FADD is an adapter in the exogenous pathway of tumour necrosis factor-induced apoptosis, and death receptor activation induces FADD recruitment, forming the death-inducing signalling complex DISC and leading to caspase-8 cleavage, thereby activating the apoptotic executor caspase-3 and ultimately causing cell death. This study identified, for the first time, that FOXO3a modulated miR-633, which directly targets FADD, and may contribute to the development of chemotherapy resistance-reversing agents that function by promoting apoptosis.

### 3.4 Effects of FOXO3a and microRNA in colorectal cancer

Colorectal cancer is a frequent malignant tumour in the gastrointestinal tract and is the third most malignant tumour in the world (52). Colorectal cancer and is prone to recurrence and metastasis (53), creating continuous clinical challenges (54). Certain epigenetic alterations, such as DNA methylation, RNA methylation, and histone acetylation, are closely associated with tumorigenesis. Dysregulation of microRNAs showing oncogenic activity is highly correlated with the development of colorectal cancer. Specifically, microRNAs induce tumour progression by regulating intracellular signalling networks, driving cell proliferation and triggering resistance to radiotherapy drugs; in contrast, microRNAs of oncogenes accelerate apoptosis, reverse chemoresistance and attenuate the condition of patients with cancer by regulating the levels of cancer-related factors.

#### 3.4.1 Altered FOXO3a levels are induced by microRNAs

By transfecting miR-493-5p mimics into colorectal cancer cell lines, we identified significantly lower levels of PI3K, AKT and FOXO3a proteins compared to those measured after transfection into control cells. The reasons for these findings, which are opposite those of previously reported outcomes, are attributed to PI3K and AKT consistently taking an active part in promoting tumour cell progression; however, the literature indicates that the expression of PI3K, AKT and FOXO3a is reduced by miR-493-5p, which is an oncogene that promotes the growth of colorectal cancer cells. It is conjectured that the overly powerful tumour-promoting role of miR-493-5p in colorectal cancer cell growth involves, which allows PI3K and AKT expression to be relatively reduced in comparison. The precise reason remains to be further analysed via experimentation (55). Resistance to radiation therapy is a contributor to poor treatment outcomes in colorectal cancer patients, and it is crucial to find markers that indicate sensitivity to radiation therapy in colorectal cancer. miR-155 has been documented as an oncogene in a variety of cancers, and the level FOXO3a, a candidate target of miR-155, was significantly reduced in colorectal cancer cell lines resistant to radiation therapy, while the miR-155 content was significantly elevated; thus, a clear negative correlation between miR-155 and FOXO3a gene expression was identified (56).MiR-96 is upregulated in colorectal cancer and promotes cell growth and proliferation, thus functioning as an oncogene. In addition, TP53INP1 and two members of the FOXO family, FOXO1 and FOXO3a, which are genes directly targeted by miR-96, were identified, and their discovery may facilitate the elucidation of the regulatory mechanism underlying miR-96 effects on colorectal carcinogenesis (57).Transfection of miR-223 inhibitors markedly upregulated FOXO3a expression, increased Bim levels, attenuated cell proliferation, and potentiated apoptosis in SW620 colorectal cancer cells (58).

#### 3.4.2 FOXO3a affects microRNA expression

The demethylase ALKBH5 decreases the m6A methylation modification rate of FOXO3a to increase its stable expression and plays a supportive role in the proliferation of colorectal cancer cells. miR-21 normally enhances colorectal cancer progression, and when FOXO3a is demethylated, miR-21 expression is downregulated, resulting in the suppression of colorectal cancer cell proliferation (59). Thus, the search for factors that can arrest FOXO3a methylation and modify FOXO3a expression may lead to better management of the pathogenic course of colorectal cancer. Evidence suggests that when GAS5 is overexpressed in colorectal tumour cells, the FOXO3a level is synergistically increased and plays an indispensable role in repressing the proliferation of colorectal tumour cells, and the addition of miR-182-5p blocks the GAS5-induced upregulation of FOXO3a protein expression (60). Therefore, the authors propose that GAS5 modulates the expression of FOXO3a, which suppresses the function of miR-182-5p and hence reduces the progression of colorectal cancer. In summary, this recent report suggests that the FOXO3a level is low in both colorectal cancer and radioresistant colorectal cancer cells and that increasing the level of FOXO3a leads to a marked therapeutic effect on colorectal cancer patients. This finding may lead to the development of a drug that increases FOXO3a expression as a way to optimize treatment efficacy and improve prognosis.

### 3.5 Effects of FOXO3a and microRNA in prostate cancer

Prostate cancer accounts for the majority of malignant tumours in males (61), with a rapidly increasing incidence and a high burden (62). Its development is correlated with gene expression levels, including changes caused by gene repression, transcription and expression, the most frequent effect of which is the regulation of microRNA in this disease. Since FOXO3a is a transcription factor with the ability to silence tumour cells, this review describes both of its regulatory effects in prostate cancer, which is informative, although the literature is not extensive.

#### 3.5.1 Variations in FOXO3a level caused by microRNA

MiR-1307 is overexpressed in prostate cancer cells and tissues, apparently fostering cell propagation and tumorigenesis when a cell cycle blockade is relieved by miR-1307 inhibition of the cell cycle suppressors p21 and p27 at both the mRNA and protein levels, and miR-1307 prevents the biological effects mediated via FOXO3a by directly binding to the 3’-UTR of FOXO3a and promoting cell proliferation in prostate cancer (63). A report clarified that miR-592 bound to the 3’-UTR of FOXO3a and changed the levels of FOXO3a-related downstream proteins p21 and cyclin D1. miR-592 was shown to downregulate the expression of p21 and upregulate cytokinesis mediated via cyclin D1. miR-592 is a cell cycle inhibitor, as mentioned in a previous study (64), and cyclin D1 is a cell cycle activator, confirming the influence of miR-592 on the regulatory effect of FOXO3a on the prostate cancer cell cycle (65). As the target genes of miR-96, FOXO1 and FOXO3a exert a protective effect against prostate cancer, while inhibition of FOXO1 and FOXO3a expression by transfected siRNAs led to an increase in the number of cancer cells. MiR-96 has been proven to be a biomarker for the early diagnosis of prostate cancer, and repression of miR-96 while restoring the behaviour of FOXO members may be a potential therapeutic target (66). MiR-96 causes tumour growth inhibition by targeting distinct downstream substrates in diseases such as nasopharyngeal carcinoma and osteosarcoma (67, 68). Its promoting effects on prostate cancer have been consistently reported in the majority of the literature. Therefore, it is concluded that the action of various microRNAs in each disease is not identical, and the targeting and regulation of diverse elements may exert various impacts on cell growth; therefore, research at the genetic level is needed to discover the majority of the unknown factors. Oxygen and nutrient supplies in peripheral vascular tissues are necessary to support tumour cell growth. Under low-oxygen conditions, cells obtain energy by inhibiting aerobic respiration and enhancing glycolysis. MiR-223-3p notably increased glucose depletion as well as lactate and ATP production in radioresistant prostate cancer cells, and the concomitant overexpression of FOXO3a attenuated miR-223-3p-induced glycolytic activation. In the presence of glycolysis inhibitors, the viability of miR-223-3p-overexpressing prostate cancer cells was substantially diminished, and the protein expression apoptosis-related factors Bax and caspase-3 were upregulated (69). These results suggest that the inhibition of glycolysis reverses miR-223-3p-induced radioresistance to some extent.

## 4 Summary and outlook

**Figure 3:**
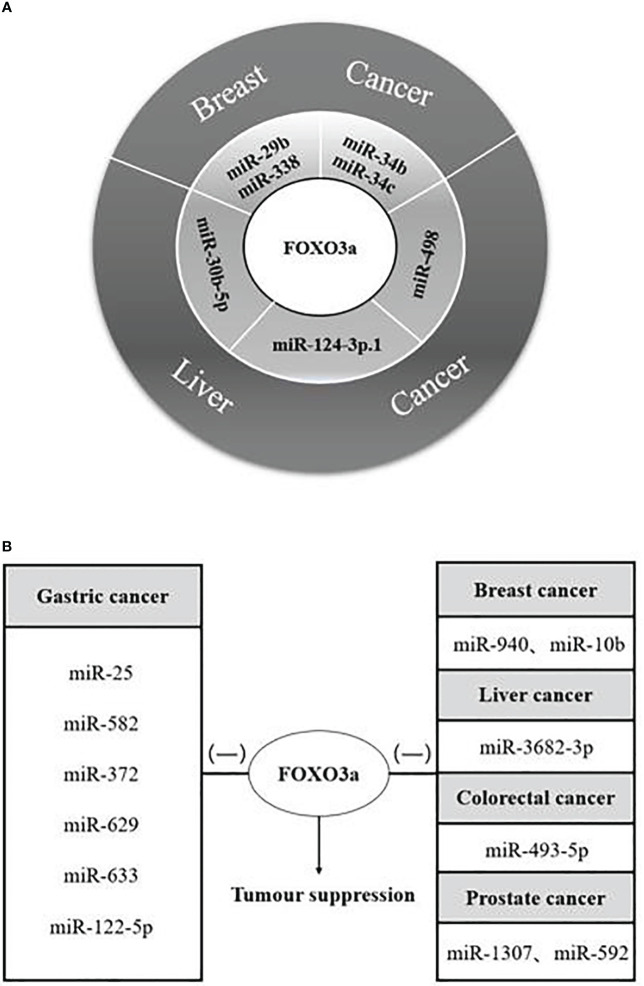
MicroRNAs synergize/antagonize FOXO3a to regulate tumour progression. **(A)** MicroRNAs synergize with FOXO3a to inhibit breast and liver cancer progression; Both microRNAs and FOXO3a are tumour suppressors. **(B)** MicroRNAs antagonism of FOXO3a to promote carcinogenesis. (-) represents antagonism with FOXO3a; FOXO3a possesses tumour suppressor function; MicroRNAs promote tumour proliferation.

Various cancer investigations identified that FOXO3a transcription factor has a significant inhibition, and its interactions with diverse microRNAs affected cancer cell proliferation, apoptosis, and cell cycle progression, but this effect has an uncertainty in hepatocellular carcinoma. When miR-485-5p and miR-498 were combined with long-chain non-coding RNAs, respectively, FOXO3a acted as a cancer-promoting agent, it is possible that the incorporation of long-stranded non-coding RNAs leads to the alteration of the SNPS site of FOXO3a, which is proved to trigger cancer or increase the risk of cancer (70–72), and the specific reasons need to be further investigated in depth. The paper concluded the results of the interactions of twenty-five microRNAs with FOXO3a in malignant tumours such as breast, liver, gastric, colorectal, and prostate cancers: There were dual microRNAs synergizing with FOXO3a to restrain the growth of breast cancer cells miR-29b, miR-338, and miR-34b, miR-34c; and a single microRNA synergizing with FOXO3a to suppress hepatocellular carcinoma cells, such as miR-30b-5p, miR-124-3p.1, and miR-498 (**Figure 3A**), the tumour suppressor function of these microRNAs makes it possible to be the target of drugs for cancer therapy.

There are twelve microRNAs antagonizing FOXO3a to promote individual tumour cell growth (**Figure 3B**), in addition to that, five microRNAs, miR-155, miR-21, miR-182-5p, miR-96, and miR-223, can antagonize FOXO3a to promote a variety of cancer progression (**Table 1**), at the same time, we discovered that the effects of a single microRNA antagonizing FOXO3a on various cancers also yielded inconsistent results. The modulation of the autophagy pathway by miR-223 with the involvement of FOXO3a intermediates re-sensitized drug-resistant cells of hepatocellular carcinoma and improved the therapeutic resistance and prognosis of hepatocellular carcinoma cells. MiR-223, however, promotes the growth of cancer cells and worsens the disease progression in colorectal carcinoma and prostate carcinoma. Therefore, further validation of the role of miR-223 in a variety of tumour cells is also required, and autophagy as a protective pathway may make the role of genes exerting tumour suppressor effects stronger. Four microRNAs, miR-155, miR-21, miR-182-5p, and miR-96, function as tumour cell growth promoters in more than two of the above malignant tumours, so we have reason to suspect that they are highly correlated with malignant tumourigenesis, and we expect that they will work as molecular markers for malignant cancer diagnosis in the future.

**Table 1:** MicroRNAs regulating multiple tumour processes.

MicroRNA	Associated tumour diseases		
MiR-182-5p	Breast cancer	Liver cancer	Colorectal cancer
MiR-155	Breast cancer	Liver cancer	Colorectal cancer
MiR-96	Gastric cancer	Colorectal cancer	Prostate cancer
MiR-21	Breast cancer	Colorectal cancer	
MiR-223	**Liver cancer**	Colorectal cancer	Prostate cancer

All the above microRNAs are antagonistic to FOXO3a but miR-223 in liver cancer, and FOXO3a inhibits the above tumour cell processes.

MicroRNA expression varies in various tumour cells, and in addition to the interaction with transcription factors such as FOXO3a, the regulation of signalling pathways is also one of the important mechanisms. Owing to the fact that microRNAs are small molecules when they work to arouse cells to make a series of responses, it is necessary for various kinase-like substances involved in the regulation of signalling pathways to gradually amplify the signals. The signalling pathways involved in the five malignant tumours include PI3K/AKT, Snail, VEGF-A/NRP1, Wnt/β-catenin, etc., which are mainly used as cell cycle promoters to cause cell proliferation by increasing the transcription of c-Myc and up-regulating the expression of nuclear genes; blocking apoptosis so that the expression of related genes is reduced, including the caspase gene family, Bcl-2, Bcl-xl, Bim, etc.; Inhibition of G1 phase kinase complexes, such as cyclin D1, results in cell cycle arrest, causing elevated expression of the cycle-dependent protein repressor p27; promotes angiogenesis and meets cellular demands for oxygen and nutrients in order to promote cancer cell proliferation, invasion and metastasis (**Table 2**). Different microRNAs influence tumour progression through post-transcriptional regulation of protein expression, and protein molecular crossovers and associations between different signalling pathways make it possible to influence disease development.

**Table 2:** Signalling regulation by microRNAs and FOXO3a.

Malignant tumours	MicroRNA↔FOXO3a	Involved proteins	regulatory process
Breast cancer	MiR-21/MiR-34b/c→FOXO3a	PTEN/AKT	proliferation
	MiR-155→FOXO3a	c-MYC	energy metabolism
	MiR-29b/338→FOXO3a	VEGF-A/NRP1	invasion and metastasis
	MiR-10b←FOXO3a	E-catenin/N-catenin	
Liver cancer	MiR-182-5p→FOXO3a	AKT/Bcl-2/Bcl-xl/Wnt/c-MYC/cyclinD1	proliferation
	MiR-3682-3p→FOXO3a	PI3K/AKT/β-catenin/c-MYC	stemness maintenance
	MiR-155→FOXO3a	Bim/caspase-3/caspase9	apoptosis
	MiR-124-3p.1→FOXO3a	AKT2/SIRT1	
Gastric cancer	MiR-582→FOXO3a	PI3K/AKT/Snail	metastasis
	MiR-25→FOXO3a	p27kip1	cell cycle progression
	MiR-633←FOXO3a	FADD/caspase-8/caspase-3	apoptosis
Colorectal cancer	MiR-493-5p→FOXO3a	PI3K/AKT/FOXO3a	proliferation
	MiR-96→FOXO3a	TP53INP1/FOXO1	
	MiR-233→FOXO3a	Bim	
Prostate cancer	MiR-1307→FOXO3a	p21/p27	proliferation
	MiR-592→FOXO3a	p21/cyclinD1	cell cycle progression
	MiR-223-3p→FOXO3a	Bax/caspase3	apoptosis

From the above summary, it is clear that the combination of multiple microRNAs will undoubtedly have a positive impact on tumour diagnosis and treatment. Meanwhile, the powerful cancer-inhibitory effect of FOXO3a is also indisputable. Therefore, clarifying the mechanism of interactions between microRNAs and FOXO3a in cancer can open up new ideas for the development of tumour-therapeutic drugs.

## Author contributions

LS: Writing – original draft, Formal Analysis. SC: Funding acquisition, Writing – review & editing. JL: Writing – review & editing, Software. DB: Software, Writing – review & editing. CH: Writing – review & editing, Resources. YZ: Writing – review & editing, Formal Analysis.
